# Synthesis and Biological Evaluation of New Nitroimidazole Derivatives as Anti-*Helicobacter pylori* Agents Against Metronidazole-Resistant Strains

**DOI:** 10.5812/ijpr-137969

**Published:** 2023-10-14

**Authors:** Zahra Bayati, Salimeh Amidi, Mahnaz Shahabimehr, Masoud Alebouyeh, Arash Mahboubi, Sayyed Abbas Tabatabai

**Affiliations:** 1 Department of Pharmaceutical Chemistry, School of Pharmacy, Shahid Beheshti University of Medical Sciences, Tehran, Iran; 2 Department of Biology, Faculty of Pharmaceutical Sciences, Islamic Azad University, Tehran, Iran; 3 Gastroenterology and Liver Diseases Research Center, Research Institute for Gastroenterology and Liver Disease, Shahid Beheshti University of Medical Sciences, Tehran, Iran; 4 Food Safety Research Center, Department of Pharmaceutics, School of Pharmacy, Shahid Beheshti University of Medical Sciences, Tehran, Iran; 5 Phytochemistry Research Center, Shahid Beheshti University of Medical Sciences, Tehran, Iran

**Keywords:** Nitroimidazole Derivatives, Metronidazole Resistance, *Helicobacter pylori*, Synthesis

## Abstract

Since several *Helicobacter pylori* strains have become resistant to metronidazole, new nitroimidazole derivatives based on metronidazole were designed and synthesized with different substituents on imidazole nitrogen. The activity of the synthesized compounds was evaluated against 20 clinically isolated metronidazole-resistant *H. pylori* strains. Some synthesized compounds were effective against those metronidazole-resistant *H. pylori* strains. Three compounds exhibited the most potent inhibitory activities (MIC_50_ = 8 µg/mL and MIC_90_ = 16 µg/mL).

## 1. Background

*Helicobacter pylori* is a gram-negative, microaerophilic bacterium. It is responsible for most cases of active chronic gastritis, peptic ulcer, intestinal metaplasia, low-grade mucosa-associated lymphoid tissue lymphoma, and/or cancer development (1). Its infection rate is about 50% worldwide, with prevalence rates ranging from 20% to more than 80% in different countries (2, 3). In gastric mucosal infection cases, eradicating *H. pylori* seems to treat ulcers and the infection (4, 5). The most effective regimen for the treatment of *H. pylori* infection is a combination of a proton pump inhibitor and 2 antimicrobial agents (mainly clarithromycin and either metronidazole [MTZ] or amoxicillin) (6).

Different methods are applied to determine the *H. pylori* susceptibility to MTZ. Nevertheless, an association between MTZ resistance and treatment failure has been found. Combination therapy with clarithromycin and MTZ led to a cure rate of 95% when the strains were susceptible to MTZ and 76% when the strains were MTZ-resistant (MIC = 8 µg/mL, as determined by the agar dilution method recommended by EUCAST) (7, 8).

Metronidazole is one of the well-known compounds of the 5-nitroimidazole class, which has been used as an antimicrobial agent for several years (9). Since derivatives with a 5-nitroimidazole nucleus have been evaluated and characterized for their toxicity and metabolism profile, this nucleus has great potential to be widely applied in drug design (10).

There is a report on the synthesis and biological evaluation of new 5-nitroimidazole derivatives. A group of 1-(2-hydroxypropyl)-2-styryl-5-nitroimidazole derivatives was synthesized, and their antibacterial activities and toxicity were evaluated (11). Metronidazole derivatives that contain piperazine were also introduced. These new compounds exhibited proper antibacterial effects against gram-positive strains (9).

Despite the unique antimicrobial spectrum of 5-nitroimidazole derivatives in the treatment of infectious diseases, especially *H. pylori* infection, there is still a great concern about *H. pylori *resistance to MTZ, which would result in the need for new active agents against MTZ-resistant *H. pylori* strains. These observations have led us to investigate novel MTZ compounds with similar structural properties to overcome the resistance problem.

**Figure 1.**
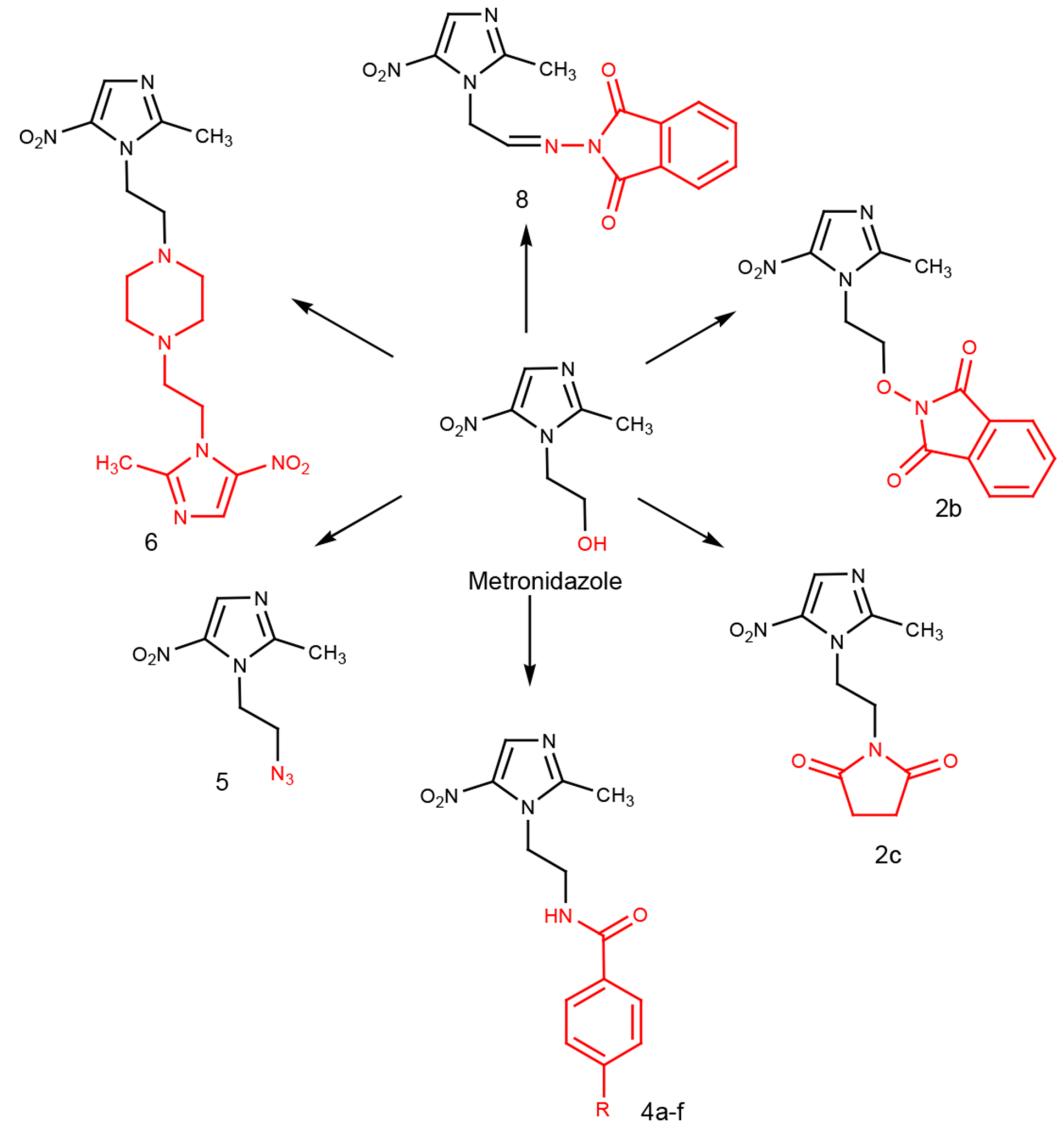
Structure of designed compounds

Following our previous research on synthesizing bioactive azole rings (12-20) and considering the biological effects of the nitroimidazole scaffold, this study focused on the design and synthesis of novel compounds containing a nitroimidazole structure. The anti-*H. pylori* effects of these compounds were evaluated against MTZ-resistant strains (Figure 1). 

## 2. Methods

### 2.1. Materials

All chemicals and reagents used in this study were commercially attained from Merck or Aldrich Companies (Germany).

For obtaining ^1^H NMR spectra, a Bruker FT-500 MHz instrument (Bruker Biosciences, USA) was used, and chemical shifts (δ) were reported as parts per million (ppm). The electrospray mass (ESI-MS) spectra of the synthesized compounds and elemental analysis were acquired using an Agilent 4610 triple quadrupole mass spectrometer and elemental analyzer (Costech, Italy), respectively. The melting points of the synthesized compounds were determined using the Electrothermal 9100 melting point apparatus. In addition, the Perkin Elmer 1420 ratio recording spectrometer was used to generate the infrared spectra.

### 2.2. Synthesis

**2-(2-(2-Methyl-5-nitro-1*H*-imidazol-1-yl)ethoxy) isoindoline-1,3-dione (2b) (** 21 **):**

To a mixture of metronidazole 1 (0.5 g, 2.92 mmol), triphenylphosphine (1.125 g, 4.28 mmol), and hydroxyphthalimide (0.7 g, 4.28 mmol) in 26 mL of dry THF, DIAD (diisopropyl azodicarboxylate) (1.9 mL, 9.75 mmol) was added in one portion at room temperature. The reaction mixture was stirred for 24 h. The solvent was removed under low pressure. The residue was triturated with diethyl ether and filtered to obtain a white solid with the following characteristics:

Yield: 77.77%; mp: 184 - 186ºC; crystallization in diethyl ether; IR (KBr, cm^-1^): 1783, 1734 (C=O),1520 (NO_2_), 1357 (NO_2_); ^1^H NMR (500 MHz, CDCl_3_): δ: 2.64 (3H, s, CH_3_), 4.60 (2H, t, J = 4.9 Hz, CH_2_-N), 4.75 (2H, t, J = 5 Hz, CH_2_-O), 7.78 (2H, m, ArH), 7.84 (2H, m, ArH), 8.07 (1H, s, Imidazole-H); ESI-MS (m/z): [M+Na]^+^ 339; Anal. Calc. for C_14_H_12_N_4_O_5_ (316.08): C 53.17, H 3.82, N 17.71; found: C 53.15, H 3.81, N 17.75.

**1-(2-(2-Methyl-5-nitro-1*H*-imidazol-1-yl)ethyl) pyrrolidine-2,5-dione (2c) (** 22 **):**

A mixture of metronidazole 1 (0.5 g, 2.92 mmol), triphenylphosphine (1.14 g, 4.34 mmol), and succinimide (0.43 g, 4.34 mmol) in 25 mL dry THF was cooled to 0 - 5ºC. Then, a solution of DIAD (diisopropyl azodicarboxylate) (1.9 mL, 9.75 mmol) in 3 mL dry THF was added dropwise for 20 min. The reaction mixture was stirred for 24 h. The progress of the reaction was monitored by TLC. The solvent was removed to give oil under low pressure. The oil was solidified using diethyl ether and then crystallized from 2-propanol. The following characteristics appeared:

Yield: 55%; mp: 151 - 153ºC; IR (KBr, cm^-1^): 1703 (C=O), 1528 (NO_2_), 1374 (NO_2_); ^1^H NMR (500 MHz, DMSO-d6): δ: 2.36 (3H, s, CH_3_), 2.52 (4H, broad s, Succinimide), 3.76 (2H, t, J = 5.9 Hz, CH_2_-Succinimide), 4.42 (2H, t, J = 5.9 Hz, CH_2_-Imidazole), 8.00 (1H, s, Imidazole-H); ESI-MS (m/z): [M+Na]^+^ 275; Anal. Calc. for C_10_H_12_N_4_O_4_ (252.09): C 47.62, H 4.80, N 22.21; found: C 47.61, H 4.79, N 22.19.

**2-(2-Methyl-5-nitro-1*H*-imidazol-1-yl)ethyl benzenesulfonate (2d):**

A solution of metronidazole 1 (10 g, 58.47 mmol) in dry pyridine was cooled in an ice bath; then, benzene sulfonyl chloride (8.1 mL, 63.8 mmol) was added dropwise. The reaction mixture was stirred for 24 h at room temperature. The achieved solution was diluted with ice/water, and the obtained product was washed several times with water, with the following characteristics:

Yield: 83%; mp: 145 - 150 ºC; IR (KBr, cm^-1^): 1520 (NO_2_), 1380 (NO_2_), 1340 (SO_2_), 1180.

**2-(2-Methyl-5-nitro-1*H*-imidazol-1-yl)ethanamine dihydrobromide (3):**

A mixture of metronidazole 1 (4 g, 23.4 mmol), triphenylphosphine (9.18 g, 35 mmol), and phthalimide (5.15 g, 35 mmol) in 200 mL dry THF was cooled to 0 - 5ºC. Then, a solution of DIAD (diisopropyl azodicarboxylate) (15.2 mL, 75 mmol) in 20 mL dry THF was added dropwise for 20 min. The reaction mixture was stirred for 1 h. The progress of the reaction was monitored by TLC. The solvent was removed to give oil under low pressure. The oil was solidified using diethyl ether and then crystallized from 2-propanol to obtain 2a. A mixture of 2a (3.02 g, 10 mmol) in 30% HBr (110 mL) was refluxed for 16 h. The acid was separated by distillation. Absolute ethanol (100 mL) was added to the mixture and dried under low pressure. The residue was washed with diethyl ether (4 × 30 mL) and crystallized from ethanol (23). It had the following characteristics:

Yield: 80%; mp: 221 - 228ºC; ^1^H NMR (500 MHz, DMSO-d_6_): δ: 2.61 (3H, s, CH3), 3.33 (2H, m, CH_2_-NH_2_), 4.60 (2H, t, J = 6.4 Hz, CH_2_-Imidazole), 8.20 (3H, NH_3_^+^), 8.43 (1H, s, Imidazole-H), 9.87 (1H, broad s, NH^+^-Imidazole); ESI-MS (m/z): [M+H]^+^ 171; Anal. Calc. for C_6_H_12_Br_2_N_4_O_2_ (329.93): C 21.71, H 3.64, N 16.88; found: C 21.73, H 3.65, N 16.91.

#### 2.2.1. General Synthesis Method for Compounds (4a-f)

Five milliliters of triethylamine was added to a solution of 3 (0.5 g) in dry THF (20 mL), and the mixture was cooled to 0 - 5ºC. Then, appropriate benzoyl chloride (2.85 mmol) was added dropwise. The mixture was stirred for 5 h. The solvent was removed under low pressure. The residue was dissolved in dichloromethane (30 mL) and extracted with acidified water by 3 M HCl (5 × 20 mL). The combined aqueous layers were made alkaline (pH 7.5 - 8) with NaOH solution and extracted with dichloromethane (5 × 20 mL). The combined organic layers were washed with a sodium chloride saturated solution (2 × 25 mL) and then dried with Na_2_SO_4_. The organic solvent was evaporated under a vacuum. The residue was crystallized from ethanol.

**N-(2-(2-Methyl-5-nitro-1*H*-imidazol-1-yl)ethyl) benzamide (4a) (** 24 **)**

It had the following characteristics: Yield: 30%; mp: 128.7 - 131.2ºC; IR (KBr, cm^-1^): 3364 (NH), 1637 (C=O), 1544 (NO_2_), 1366 (NO_2_); ^1^H NMR (500 MHz, CDCl_3_): δ: 2.52 (3H, s, CH_3_), 3.83 (2H, m, CH_2_-NH), 4.63 (2H, t, J = 6.2 Hz, Imidazole-CH_2_), 6.76 (1H, t, NH), 7.46 (2H, t, J = 7.63 Hz, ArH), 7.55 (1H, t, J = 7.4 Hz, ArH), 7.75 (2H, d, J = 7.3 Hz, ArH), 7.97 (1H, s, Imidazole-H); ESI-MS (m/z): [M+Na]^+^ 297; Anal. Calc. for C_13_H_14_N_4_O_3_ (274.11): C 56.93, H 5.14, N 20.43; found: C 56.92, H 5.15, N 20.42.

**4-Fluoro-N-(2-(2-methyl-5-nitro-1*H*-imidazol-1-yl) ethyl)benzamide (4b):**

It had the following characteristics: Yield: 35%; mp: 202 - 204ºC; IR (KBr, cm^-1^); 3386 (NH), 1648 (C=O), 1556 (NO_2_), 1375 (NO_2_); ^1^H NMR (500 MHz, CDCl_3_): δ: 2.27 (3H, s, CH_3_), 3.54 (2H, m, CH_2_-NH), 4.37 (2H, t, J = 6.1 Hz, Imidazole-CH_2_), 6.89 (2H, t, J = 8.6 Hz, ArH), 7.65 (2H, d, J = 8.6 Hz, ArH), 7.78 (1H, s, Imidazole-H), 8.23 (1H, t, NH); ESI-MS (m/z): [M+Na]^+^ 315; Anal. Calc. for C_13_H_13_FN_4_O_3_ (292.10): C 53.42, H 4.48, N 19.17; found: C 53.44, H 4.48, N 19.18.

**4-Chloro-N-(2-(2-methyl-5-nitro-1*H*-imidazol-1-yl) ethyl)benzamide (4c):**

It had the following characteristics: Yield: 45%; mp: 197.2 - 199.2ºC; IR (KBr, cm^-1^): 3348 (NH), 1639 (C=O), 1543 (NO_2_), 1356 (NO_2_); ^1^H NMR (500 MHz, DMSO-d6): δ: 2.35 (3H, s, CH_3_), 3.63 (2H, m, CH_2_-NH), 4.44 (2H, t, J = 5.8 Hz, Imidazole-CH_2_), 7.53 (2H, d, J = 8.8 Hz, ArH), 7.74 (2H, d, J = 7.8 Hz, ArH), 8.02 (1H, s, Imidazole-H), 8.76 (1H, t, J = 5.82 Hz, NH); ESI-MS (m/z): [M+Na]^+^ 331.5; Anal. Calc. for C_13_H_13_ClN_4_O_3_ (308.07): C 50.58, H 4.24, N 18.15; found: C 50.57, H 4.24, N 18.16.

**N-(2-(2-Methyl-5-nitro-1*H*-imidazol-1-yl)ethyl)-4- nitrobenzamide (4d):**

It had the following characteristics: Yield: 32%; mp: 197.8 - 199.3ºC; IR (KBr, cm^-1^): 3425 (NH), 1666 (C=O), 1514 (NO_2_), 1361 (NO_2_); ^1^H NMR (500 MHz, DMSO-d6): δ: 2.36 (3H, s, CH_3_), 3.67 (2H, m, CH_2_-NH), 4.46 (2H, t, J = 5.7 Hz, Imidazole-CH_2_), 7.96 (2H, d, J = 8.7 Hz, ArH), 8.05 (1H, t, J = 8.7 Hz, NH), 8.31 (2H, d, J = 8.6 Hz, ArH), 9.02 (1H, s, Imidazole-H); ESI-MS (m/z): [M+H]^+^ 320, [M+Na]^+^ 342; Anal. Calc. for C_13_H_13_N_5_O_5_ (319.09): C 48.90, H 4.10, N 21.94; found: C 49.01, H 4.10, N 21.93.

**4-Methyl-N-(2-(2-methyl-5-nitro-1*H*-imidazol-1-yl) ethyl)benzamide (4e):**

It had the following characteristics: Yield: 37%; mp: 187 - 191ºC; IR (KBr, cm^-1^): 3368 (NH), 1637 (C=O), 1543 (NO_2_), 1353 (NO_2_); ^1^H NMR (500 MHz, DMSO-d6): δ: 2.33 (3H, s, phenyl-CH_3_), 2.50 (3H, s, Imidazole-CH_3_), 3.62 (2H, m, CH_2_-NH), 4.43 (2H, t, J = 5.9 Hz, Imidazole-CH_2_), 7.25 (2H, d, J = 8.0 Hz, ArH), 7.63 (2H, d, J = 8.1 Hz, ArH), 8.02 (1H, s, Imidazole-H), 8.58 (1H, t, J = 5.3 Hz, NH); ESI-MS (m/z): [M+H]^+^ 289; Anal. Calc. for C_14_H_16_N_4_O_3_ (288.12): C 58.32, H 5.59, N 19.43; found: C 58.37, H 5.59, N 19.42.

**4-Methoxy-N-(2-(2-methyl-5-nitro-1*H*-imidazol-1-yl) ethyl)benzamide (4f):**

It had the following characteristics: Yield: 25%; mp: 149.2 - 150.3ºC; IR (KBr, cm^-1^): 3368 (NH), 1642 (C=O), 1523 (NO_2_), 1370 (NO_2_); ^1^H NMR (500 MHz, CDCl_3_): δ: 2.52 (3H, s, CH_3_), 3.77 (2H, m, CH_2_-NH), 3.87 (3H, s, OCH_3_), 4.62 (2H, t, J = 6.2 Hz, Imidazole-CH_2_), 6.62 (1H, t, J = 8.8 Hz, NH), 6.95 (2H, d, J = 8.8 Hz, ArH), 7.73 (2H, d, J = 6.9 Hz, ArH), 7.97 (1H, s, Imidazole-H); ESI-MS (m/z): [M+Na]^+^ 327; Anal. Calc. for C_14_H_16_N_4_O_4_ (304.12): C 55.26, H 5.30, N 18.41; found: C 55.30, H 5.29, N 18.40.

**1-(2-Azidoethyl)-2-methyl-5-nitro-1*H*-imidazole (5) (** 25 **):**

A mixture of 2d (1 g, 3.58 mmol), sodium azide (35.8 mmol), triethylamine in water (5 mL), and 18-crown-6-ether in xylene (30 mL) was refluxed for 48 h. The solvent was removed by distillation. The residue was diluted in water and extracted with ethyl acetate (4 × 20 mL). The combined organic layers were evaporated. The residue was triturated with n-hexan. The resulting solid was washed several times with n-hexane, which gave the following characteristics:

Yield: 54%; mp: 50 - 53ºC; IR (KBr, cm^-1^): 2123 (N_3_), 1540 (NO_2_), 1372 (NO_2_); ^1^H NMR (500 MHz, DMSO-d6): δ: 2.48 (3H, s, CH_3_), 3.79 (2H, t, J = 5.6 Hz, CH_2_-N_3_), 4.48 (2H, t, J = 5.6 Hz, Imidazole-CH_2_), 8.03 (1H, s, Imidazole-H); ESI-MS (m/z): [M+H]^+^ 197; Anal. Calc. for C_6_H_8_N_6_O_2_ (196.07): C 36.74, H 4.11, N 42.84; found: C 36.65, H 4.12, N 42.87.

**1,4-Bis(2-(2-methyl-5-nitro-1*H*-imidazol-1-yl)ethyl) piperazine (6):**

A mixture of 2d (1 g, 3.58 mmol), piperazine (0.092 g, 1.06 mmol), and sodium bicarbonate (excess) in dioxane was refluxed for 24 h. The solvent was evaporated under low pressure. Water (10 mL) was added to the residue and extracted by chloroform (3 × 10 mL). The combined organic layers were washed with water, dried with Na_2_SO_4_, and purified using column chromatography (mobile phase first: chloroform, Second: chloroform 83.3%: methanol 16.6%), giving the following characteristics:

Yield: 25%; mp: 197 - 199ºC; ^1^H NMR (500 MHz, CDCl_3_): δ: 2.47 (8H, broad s, Piperazine), 2.53 (6H, s, CH_3_), 2.67 (4H, t, J = 5.9 Hz, CH_2_-N), 4.40 (4H, t, J = 5.9 Hz, CH_2_-Imidazole), 7.94 (2H, s, Imidazole-H); ESI-MS (m/z): [M+Na]^+^ 415; Anal. Calc. for C_16_H_24_N_8_O_4_ (392.19): C 48.97, H 6.16, N 28.56; found: C 48.94, H 6.17, N 28.53.

**2-(2-Methyl-5-nitro-1*H*-imidazol-1-yl)acetaldehyde (7):**

Oxalyl chloride (2.3 mL, 25.3 mmol) was added dropwise to 160 mL of dry dichloromethane under argon. After that, the reaction mixture was cooled to -50ºC in dry ice. Then, DMSO (17 mL, 240 mmol) was added dropwise. After 20 min, a solution of metronidazole 1 (3 g, 17.54 mmol) in 15 mL DMSO was added dropwise. By passing another 20 min, dry triethylamine (33 mL, 240 mmol) was added dropwise. The reaction was stirred for 10 minutes and then warmed to room temperature. The mixture was diluted in ethyl acetate and extracted with water (3 × 50 mL). The combined aqueous layers were extracted with ethyl acetate (3 × 250 mL). The combined organic layers were washed with saturated NaCl and dried with Na_2_SO_4_. The organic solvent was evaporated under a vacuum. The obtained residue was purified using column chromatography (mobile phase: Dichloromethane 97%: Methanol 3%) to obtain the product as an oil (26) with the following characteristics:

Yield: 68%; ESI-MS (m/z): [M+H]^+^ 170.

**2-(2-(2-Methyl-5-nitro-1*H*-imidazol-1-yl) ethylideneamino)isoindoline-1,3-dione (8):**

A mixture of acidified (acetic acid pH = 4 - 4.5) N-aminophthalimide (0.35 g, 2.16 mmol) in dioxane was added dropwise to a solution of 7 (0.37 g, 2.16 mmol) in dioxane at 60ºC, and the reaction mixture was refluxed for 19 h at 60ºC. The solvent was evaporated, and the obtained liquid was triturated with diethyl ether and filtered to obtain a brown solid with the following characteristics:

Yield: 55.2%; mp: 206 - 208ºC, crystallized in diethyl ether; IR (KBr, cm^-1^): 1785 (C=O), 1734 (C=O), 1533 (NO_2_), 1358 (NO_2_); ^1^H NMR (500 MHz, CDCl_3_): δ: 2.59 (3H, s, CH_3_), 5.36 (2H, d, J = 4.0 Hz, CH_2_-Imidazole), 7.82 (2H, m, ArH), 7.93 (2H, m, ArH), 8.03 (1H, s, Imidazole-H), 9.04 (1H, t, J = 4.0 Hz, HC = N); Anal. Calc. for C_14_H_11_N_5_O_4_ (313.08): C 53.68, H 3.54, N 22.36; found: C 53.67, H 3.53, N 22.35.

### 2.3. Biological Activity

#### 2.3.1. Helicobacter pylori Strains

As previously described, 20 clinical isolates recovered from 96 adult patients who were referred to the Digestive Endoscopy Unit of Imam Khomeini Hospital in Tehran, Iran, from 2013 to 2014 were used in this study (8).

#### 2.3.2. Susceptibility Testing

Susceptibility of the bacterial strains was determined based on the minimum inhibitory concentration (MIC) assayed by the agar twofold serial dilutions method according to the European Committee on Antimicrobial Susceptibility Testing (EUCAST) (7, 8).

The concentrations of metronidazole (MAST, London, United Kingdom) and the tested compounds ranging from 0.016 to 512 μg/mL were made in Muller Hinton agar medium (MHA, 45ºC, Merck, Germany) supplemented with 5% of sheep blood. Five microliters of fresh normal saline suspension with turbidity corresponding to 2 McFarland's standard of each clinically isolated *H. pylori* strain were inoculated on the agar medium. The inoculated plates were incubated in 5% CO_2_ for 72 hours at 37ºC (8).

*Helicobacter pylori* strain RIGLD 245 was used as a reference strain for all the experiments. The MIC was considered the lowest concentration inhibiting the bacterial visible growth. The EUCAST clinical breakpoint of > 8 µg/mL was defined for the metronidazole-resistant strains (7, 8). The results were expressed as the MICs (Table 1). The MIC_50_ and MIC_90_, corresponding to the concentrations of the synthesized derivatives inhibiting the 50% and 90% growth of the isolates, respectively, are reported in Table 2. 

## 3. Results and Discussion

### 3.1. Chemistry

**Figure 2.**
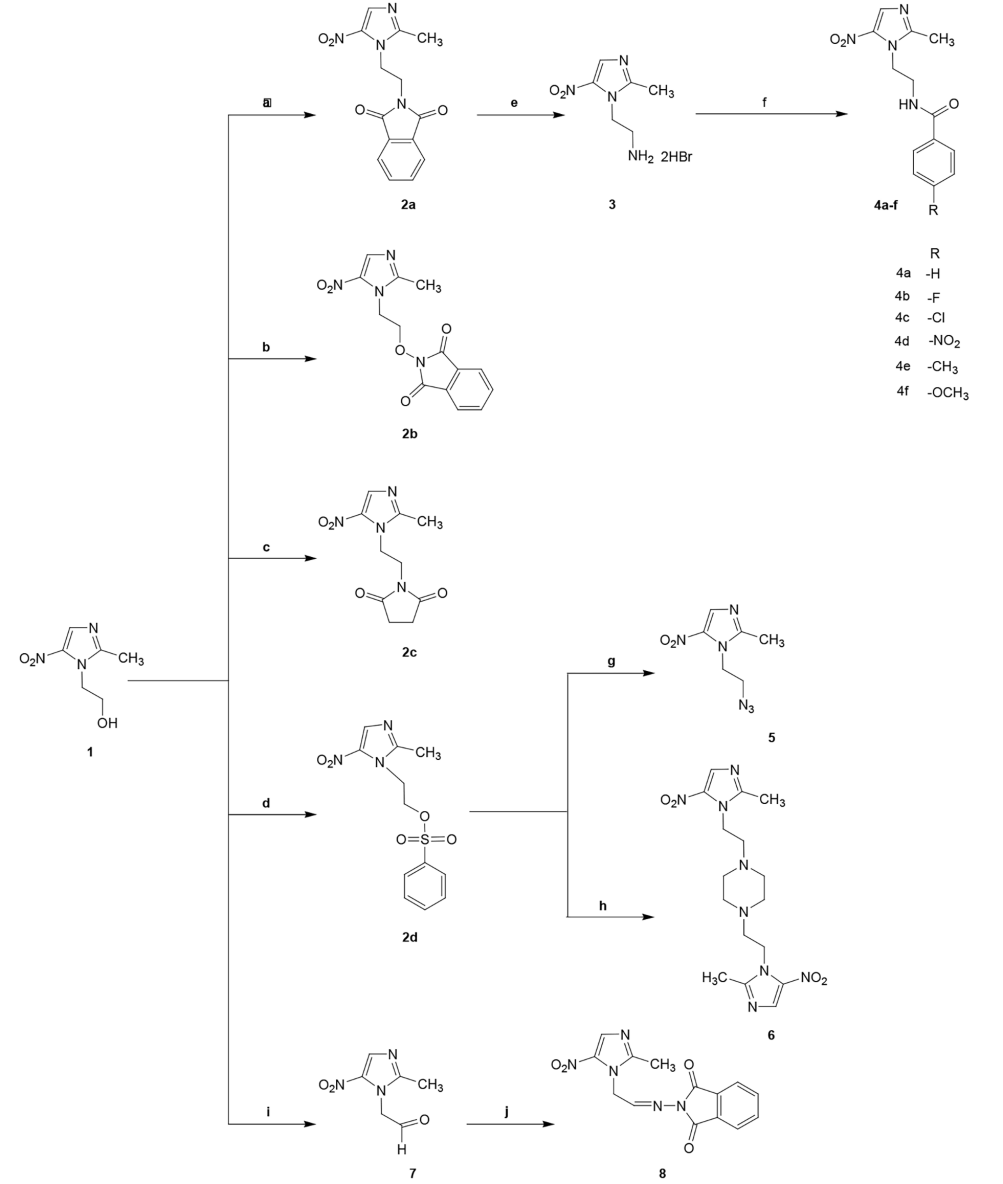
Reagents and conditions: A, Phthalimide, PPh_3_, DIAD, dry THF, Stir at rt; B, Hydroxyphthalimide, PPh_3_, DIAD, dry THF, Stir at rt; C, Succinimide, PPh_3_, DIAD, dry THF, Stir at 0ºC; D, Benzenesulfonyl chloride, dry pyridine, stir at rt; E, HBr, Reflux 16 h; F, Benzoyl chloride derivatives, dry THF, Et_3_N, stir 5 h; G, NaN_3_, Xylene, crown ether, water, Et_3_N, reflux 48 h; H, Piperazine, NaHCO_3_, reflux 24 h; I, Oxalyl chloride, DMSO, Et_3_N, stir at -50ºC; J, N-aminophthalimide, dioxane, acetic acid, reflux.

The designed compounds were derived from metronidazole, as shown in Figure 2. Compounds 2a - c were synthesized through the Mitsunobu reaction by metronidazole reacting with three different imides (phthalimide, hydroxy phthalimide, and succinimide) in the presence of triphenylphosphine (PPh3) and diisopropyl azodicarboxylate (DIAD) in dry THF. In all of these reactions, DIAD was added to a mixture of metronidazole, triphenylphosphine, and corresponding imides at 0 - 5ºC, except for hydroxyphthalimide, where DIAD was added to the mixture at room temperature in one part.

Compounds 4a - f were amide derivatives of 5-nitroimidazole and were obtained after a reaction between compound 3 and appropriate benzoyl chloride in dry THF.

The benzene sulfonate derivative of metronidazole (2d) was obtained after a reaction between metronidazole (1) and benzene sulfonyl chloride in dry pyridine. Compound 2d was reacted with sodium azide to obtain compound 5. Crown ether as a phase transfer catalyst was used in this reaction to increase sodium azide's solubility. Compound 6 was obtained by the reaction of 2d with piperazine at a 3.6/1 ratio.

The structures of the synthesized compounds were characterized by IR, ^1^H-NMR, and ESI-MS.

### 3.2. Biological Activity

The synthesized compounds were evaluated for their inhibitory activity against 20 clinically isolated *H. pylori* strains resistant to MTZ by determination of MIC, MIC_50_, and MIC_90_. The results are shown in Tables 1 and 2. 

**Table 1.** Minimum Inhibitory Concentration (µg/mL) of Metronidazole and Novel Nitroimidazole Derivatives Determined by Agar Dilution (n = 3) Against Clinically Isolated Metronidazole-resistant *Helicobacter pylori* Strains

Compounds; Strains	1 Metronidazole	2b	2c	4a	4b	4c	4d	4e	4f	5	6	8
**1**	64	8	8	8	8	8	ND	8	8	8	8	ND
**2**	64	4	4	4	4	4	ND	4	4	4	4	ND
**3**	64	8	8	8	8	8	ND	8	8	8	8	ND
**4**	64	4	4	4	4	4	ND	4	4	4	4	ND
**5**	128	16	32	16	16	16	ND	8	16	8	8	ND
**6**	128	16	32	32	16	16	ND	8	16	8	8	ND
**7**	256	32	32	32	32	32	ND	16	32	16	16	ND
**8**	256	32	32	32	32	32	ND	8	32	8	16	ND
**9**	256	32	16	32	8	32	ND	16	32	8	16	ND
**10**	256	16	16	16	16	16	ND	8	32	8	8	ND
**11**	512	32	32	32	32	32	ND	16	16	16	16	ND
**12**	512	32	16	16	32	16	ND	16	16	16	8	ND
**13**	512	16	8	32	8	32	ND	8	8	8	8	ND
**14**	512	64	16	16	32	32	ND	16	32	16	16	ND
**15**	512	16	32	32	32	32	ND	16	32	16	16	ND
**16**	512	64	32	32	32	8	ND	16	32	16	16	ND
**17**	512	8	8	8	8	8	ND	8	8	16	8	ND
**18**	512	32	32	8	32	32	ND	16	32	16	16	ND
**19**	>512	128	64	64	128	64	ND	32	64	32	32	ND
**20**	>512	128	64	64	64	64	ND	32	128	32	64	ND

Abbreviation: ND, not determined.

**Table 2.** Minimum Inhibitory Concentration of Metronidazole and Novel Nitroimidazole Derivatives Against 20 Clinically Isolated *Helicobacter pylori* Strains

Compounds	MIC_50_ (µg/mL)	MIC_90_ (µg/mL)
**1 (metronidazole)**	256	512
**2b**	16	64
**2c**	16	32
**4a**	16	32
**4b**	16	32
**4c**	16	32
**4d**	ND	ND
**4e**	8	16
**4f**	16	32
**5**	8	16
**6**	8	16
**8**	ND	ND

Abbreviation: ND, not determined.

As presented in Tables 1 and 2, almost all synthesized compounds were effective against metronidazole-resistant *H. pylori* strains. The para methyl derivative (4e) was identified as the most active compound (MIC_50_ = 8 μg/mL) against metronidazole-resistant *H. pylori* strains amongst benzoyl metronidazole derivatives (4a-f). Compounds 5 and 6 with azide and piperazine substituents exerted the highest inhibitory activity against metronidazole-resistant *H. pylori* strains. The obtained MIC_90_ values of these compounds were 16 μg/mL, which is 32-fold over the metronidazole.

## 4. Conclusions

Some nitroimidazole analogs were synthesized, and their inhibitory activity against 20 clinically isolated *H. pylori* strains resistant to metronidazole was evaluated by determining MIC_50_ and MIC_90_. Compounds 4e, 5, and 6 were the most active compounds, with the MIC_90_ of 8 μg/mL and MIC_90_ of 16 μg/mL.
